# Establishment and analysis of a novel diagnostic model for systemic juvenile idiopathic arthritis based on machine learning

**DOI:** 10.1186/s12969-023-00949-x

**Published:** 2024-01-19

**Authors:** Pan Ding, Yi Du, Xinyue Jiang, Huajian Chen, Li Huang

**Affiliations:** 1 Department of Medical Record Statistics, Wenzhou People’s Hospital, Wenzhou, China; 2 Lianyungang Maternal and Child Health Hospital, Lianyungang, China; 3 Zhoushan Center for Disease Control and Prevention, Zhoushan, China; 4 School of Public Health and Management, Wenzhou Medical University, Wenzhou, China

**Keywords:** Systemic juvenile idiopathic arthritis, Machine learning, Random forest, GEO, Diagnostic model

## Abstract

**Background:**

Systemic juvenile idiopathic arthritis (SJIA) is a form of childhood arthritis with clinical features such as fever, lymphadenopathy, arthritis, rash, and serositis. It seriously affects the growth and development of children and has a high rate of disability and mortality. SJIA may result from genetic, infectious, or autoimmune factors since the precise source of the disease is unknown. Our study aims to develop a genetic-based diagnostic model to explore the identification of SJIA at the genetic level.

**Methods:**

The gene expression dataset of peripheral blood mononuclear cell (PBMC) samples from SJIA was collected from the Gene Expression Omnibus (GEO) database. Then, three GEO datasets (GSE11907-GPL96, GSE8650-GPL96 and GSE13501) were merged and used as a training dataset, which included 125 SJIA samples and 92 health samples. GSE7753 was used as a validation dataset. The limma method was used to screen differentially expressed genes (DEGs). Feature selection was performed using Lasso, random forest (RF)-recursive feature elimination (RFE) and RF classifier.

**Results:**

We finally identified 4 key genes (ALDH1A1, CEACAM1, YBX3 and SLC6A8) that were essential to distinguish SJIA from healthy samples. And we combined the 4 key genes and performed a grid search as well as 10-fold cross-validation with 5 repetitions to finally identify the RF model with optimal mtry. The mean area under the curve (AUC) value for 5-fold cross-validation was greater than 0.95. The model’s performance was then assessed once more using the validation dataset, and an AUC value of 0.990 was obtained. All of the above AUC values demonstrated the strong robustness of the SJIA diagnostic model.

**Conclusions:**

We successfully developed a new SJIA diagnostic model that can be used for a novel aid in the identification of SJIA. In addition, the identification of 4 key genes that may serve as potential biomarkers for SJIA provides new insights to further understand the mechanisms of SJIA.

**Supplementary Information:**

The online version contains supplementary material available at 10.1186/s12969-023-00949-x.

## Introduction

Juvenile idiopathic arthritis (JIA) is a general term for arthritis of unknown etiology that lasts more than 6 weeks and develops before the age of 16 years. It is one of the most common chronic inflammatory rheumatic diseases of childhood [1–3]. Systemic juvenile idiopathic arthritis (SJIA) is currently classified as a subtype of JIA [4]. SJIA is a form of childhood arthritis with clinical features [5, 6] such as fever, lymphadenopathy, arthritis, rash and serosal disease, and even in the absence of ongoing disease activity, the consequences are not limited to joint destruction, but can also affect vision, impact overall growth, osteoporosis and underlying cardiovascular disease, leading to high rates of disability and mortality in children [1, 5, 7–9]. Although SJIA has characteristic clinical signs such as fever, a rash associated with fever and joint involvement, the early clinical manifestations are varied and some of the symptoms can be mild or atypical, making recognition and diagnosis difficult. Our treatment choices are scarce for this pediatric condition. Effective identification and intervention can reduce and prevent treatment difficulties after the disease has become severe.

Due to the complex pathogenesis of SJIA, it has been reported that SJIA can be better diagnosed through the study of biomarkers [10, 11]. In order to effectively identify high-risk patients and prevent or lessen the development of SJIA, biomarkers can be used to diagnose and treat SJIA rather than relying solely on its clinical aspects [12]. Significant progress has been achieved in the pathophysiology and treatment of SJIA in recent years, and big data research that combine genomic data and immunophenotypes may reveal new information about SJIA [13]. Just in the last few years, biologics have emerged as a promising new approach to treating SJIA [14–17]. Therefore, there is an urgent need for more accurate risk identification and treatment targeting for SJIA.

The rapid development of transcriptome sequencing technology has provided a reliable method to decipher the genetic information of diseases. Besides, it also provides a strong basis for the diagnosis and targeted treatment of various diseases [18–20]. Although some genes have been identified as genetic risk markers for SJIA [21, 22], their individual identification is limited due to the complexity and association of the genetic architecture. Importantly, the combination of multiple biomarkers to identify disease has been shown to significantly improve identification. Mathematical models suggest that genetic risk genes play important roles in identifying high-risk individuals, as well as improving the diagnostic accuracy and targeted drug development [23–25].

Peripheral blood mononuclear cell (PBMC) plays a critical role in the pathogenesis of SJIA by producing pro-inflammatory cytokines, recruiting other immune cells to the sites of inflammation, and activating the complement system. So our study is based on PBMC from SJIA children and the blood samples are easy to obtain and manipulate.

Based on 4 key genes (ALDH1A1, CEACAM1, YBX3 and SLC6A8) screened in the Gene Expression Omnibus (GEO) database, we developed a novel SJIA diagnostic model at the transcriptome level. Three methods, including least absolute shrinkage and selection operator (Lasso), random forest (RF)-recursive feature elimination (RFE) and RF classifier scores, were used together to determine the most important genes for SJIA classification. The SJIA diagnostic model based on 4 key genes was then built by selecting the RF of the optimal mtry on account of the 10-fold cross-validation with 5 repetitions and grid search. We evaluated the performance of the diagnostic model using 5-fold cross-validation as well as using an external independent validation dataset to confirm its accuracy and discriminatory power. We also investigated the expression of 4 key genes in three other isoforms of JIA (enthesitis-related arthritis, persistent oligoarthritis and rheumatoid factor negative polyarthritis) to find similarities and differences. Therefore, our study provides new insights into potential biomarkers for follow-up studies of SJIA, as well as a new predictive aid in terms of predicting SJIA at the genetic level in clinical practice.

## Materials and methods

### Data selection and processing

All datasets for this study were obtained from the GEO database, which stores information on the expression of genes using high-throughput methods. It was created by the National Center for Biotechnology Information (NCBI). The study was extensively searched through the NCBI database platform using the keyword “systemic juvenile idiopathic arthritis”. The type of datasets we chose was expression profiling by array, the type of organisms was homo sapiens, and the tissue selection of peripheral blood mononuclear cells (PBMCs). We used the MAS5.0 signal intensity as the expression level of the gene in order to unify the probe signal intensity. Datasets of MAS5.0 signal strength (GSE11907-GPL96 and GSE8650-GPL96) were downloaded in the Series Matrix File(s) of the respective GEO data. For the remaining datasets (GSE7753, GSE13501, GSE20307 and GSE21521), we downloaded the original CEL files generated by Affymetrix from GEO. The raw CEL files were processed separately using the MAS 5.0 algorithm implemented in the R package affy (version 1.72.0). Then, we converted the probe IDs to gene symbols based on the corresponding platform annotation files for the respective datasets. If multiple probes correspond to the same gene symbol, the signal average of multiple probes corresponding to the same gene symbol is calculated as the expression level of the corresponding gene. Batch correction was performed by applying the ComBat function in the R package sva (version 3.42.0) as a way to eliminate potential multicenter batch effects between different experiments and to output the correction results.

We merged and batch corrected GSE11907-GPL96, GSE8650-GPL96 and GSE13501 as the training dataset. GSE7753 was used as the external validation dataset. Details about six datasets are shown in Supplementary Table S1.

### Screening for differentially expressed genes (DEGs)

To screen out DEGs, we performed differential expression analysis in the training dataset using the traditional Bayesian approach of the R package limma (version 3.50.1). False discovery rate (FDR) < 0.05 and log2 fold change (log2FC) with absolute value > 1 were used as significance criteria for DEGs. We plotted the volcano map and the heat map to represent the expression levels of DEGs, which were created using the R packages pheatmap (version 1.0.12) and ggplot2 (version 3.3.6), respectively.

### Analysis of gene ontology and pathway enrichment

DEGs were interpreted using Gene Ontology (GO) and the Kyoto Encyclopedia of Genes and Genomes (KEGG). In this study, we used the enrichGO and enrichKEGG functions of the R package clusterProfiler (version 4.4.4) to perform GO and KEGG analyses, respectively, where GO analyses include biological processes (BP), cellular components (CC), and molecular functions (MF). We used bar graphs to show them. In addition, we again performed a gene set enrichment analysis (GSEA) using the gseKEGG function of the R package clusterProfiler to identify pathway differences between the SJIA and healthy groups. Pathways with *p* < 0.05 were considered significantly enriched.

### Assessing the role of immunomodulation

Since SJIA has historically been associated with the immune system, we aimed to explore the potential immune relationship between SJIA and healthy samples. We used the CIBERSORT algorithm of the R package cibersort (version 0.1.0) and its self-contained LM22 gene signature to quantify the fraction of each immune cell in each sample. CIBERSORT, a deconvolution algorithm, allows a reliable quantification of sensitivity and specificity distinctions and abundance of 22 human immune cell phenotypes based on transcriptomic data. The abundance of immune cells was used to explore whether there were immune differences between SJIA and healthy groups.

### Feature selection

To screen for key biomarkers, we applied machine learning in biology to perform reliable gene-based feature selection. First, we used the R package glmnet (version 4.1-3) for Lasso regression. Lasso regression provides a new feature selection algorithm that can solve the covariance problem and filter out representative variable features. A refined model is constructed using the Lambda value at the minimum binomial deviation as a criterion. Second, the RFE method combined with the RF classifier was used for feature selection using the R package caret (version 6.0–91), where the control parameters were set to a 10-fold cross-validation with 5 repetitions as well as accuracy was used as a screening criterion. The recursive feature elimination method is to train the model on the original features, and each feature gets a weight. After that, those features that have the smallest absolute value weights are kicked out of the feature set. This is recursively done until the number of remaining features reaches the desired number of features. Finally, we constructed RF models with optimal mtry parameters based on candidate genes using the R package caret and the R package randomForest (version 4.7–1.1) with control parameters set to 10-fold cross-validation with 5 repetitions. The parameter mtry denotes the number of variables randomly sampled in constructing decision tree branches in random forest modeling. Using a RF classifier, we assigned importance scores to feature genes, and we designated genes with importance scores > 80 as the key genes we need.

### Establishing an optimal SJIA classification model using random forest

Because the simple construction of random forest with default parameters could not determine whether the model was the best model, we needed to adjust the parameters of the random forest model to find the best model. The feature genes tested during feature selection were initially included to the SJIA random forest prediction model. The best parameter mtry for fitting the dataset was then found using a grid search strategy with the caret and randomForest packages. Immediately afterwards, the model was optimized on the basis of accuracy and overfitting was reduced through multiple rounds of training with 10-fold cross-validation with 5 repetitions. Finally, the SJIA random forest prediction model with optimal parameter mtry was constructed. Finally, the SJIA random forest prediction model was built with the optimal parameter mtry.

### Validating the robustness of the model using cross validation

We applied the best model to the training dataset for 5-fold cross-validation to determine the robustness of the model. Using the R package pROC (version 1.18.0), we calculated the area under the curve (AUC) of the receiver operating characteristic (ROC) curve. The accuracy, Kappa, sensitivity and specificity of the results were calculated by the confusionMatrix function of R package caret. Accuracy is the ratio of the sample size that was correctly predicted over all the sample sizes that participated in the prediction. Kappa is a metric used for consistency testing and can also be used to measure the effectiveness of classification. In the range of -1 to 1, higher values indicate better model performance. Sensitivity, also known as true-positive rate, is the percentage of actual disease correctly diagnosed by that diagnostic criterion. Specificity, also known as true-negative rate, is the percentage of actual absence of disease correctly diagnosed by that diagnostic criterion.

### Verification using validation datasets

The validity of the SJIA random forest diagnostic model was verified in an external independent validation dataset (GSE7753). Since there are multiple types of JIA, we also explored whether there are dissimilarities among the three subtypes of enthesitis-related arthritis, persistent oligoarthritis and rheumatoid factor negative polyarthritis for differential analysis of these 4 key genes. The above disease datasets were obtained from GSE13501. Details of the above three subtypes are shown in Supplementary Table S2.

To further explore the breadth of the model. We examined the predictive power of the model for other diseases, such as Systemic Lupus Erythematosus (SLE), S. aureus, S. pneumoniae, E. coli and Influenza A. We collected the GSE8650-GPL96 dataset for model performance checking of SLE and the GSE6269-GPL96 dataset for model performance checking of S. aureus, S. pneumoniae, E. coli and Influenza A. Details of the above diseases are shown in Supplementary Table S3.

### Single-cell RNA-Seq data processing

Single-cell RNA-seq data were obtained from the GSE207633 database, from which we obtained patient clinical information (from the file “GSE207633_Patient_Clinical-MetaData.xlsx”), publisher Single-cell clustering information containing cell name, patient ID, cell type, UMAP clustering information, etc. (from the file “GSE207633_ScRNASeq-MetaData.xlsx”) and single-cell RNA-seq sequencing data (from the file “GSE207633_RAW.tar”). First, we used R package hdf5r to input the single-cell RNA-seq sequencing data extracted from “GSE207633_RAW.tar” of 26 patients, and created them into Seurat objects using the CreateSeuratObject function of R package Seurat, and then merged them. CreateSeuratObject function parameters were selected by default. Next, we used the NormalizeData function of the R package Seurat to divide the read counts for each cell by the total counts for that cell, multiply by a scaling factor (10,000), and then perform the natural logarithm transformation. We then extracted the expression matrix of YBX3 in individual cells using the FetchData function of the R package Seurat and filtered out the cells that possessed the single-cell clustering information. Finally, we used single-cell clustering information for single-cell map visualization as well as mapping YBX3 expression levels in individual cells to single-cell map.

### Statistical analysis

All statistical analyses were performed with R software (version 4.1.3). The two continuous variables were compared using the Wilcoxon two-sample test. In order to assess the correlation between two continuous variables, the Spearman correlation coefficient was used. The AUC is used to test the predictive ability of the model, 0.5<AUC<0.7 for poor model performance, 0.7 ≤ AUC<0.8 for fair model performance, 0.8 ≤ AUC<0.9 for good model performance, and AUC ≥ 0.9 for excellent model performance. All tests were two-sided, and *p* < 0.05 was considered significant.

## Results

### Study Design

**Fig. 1:**
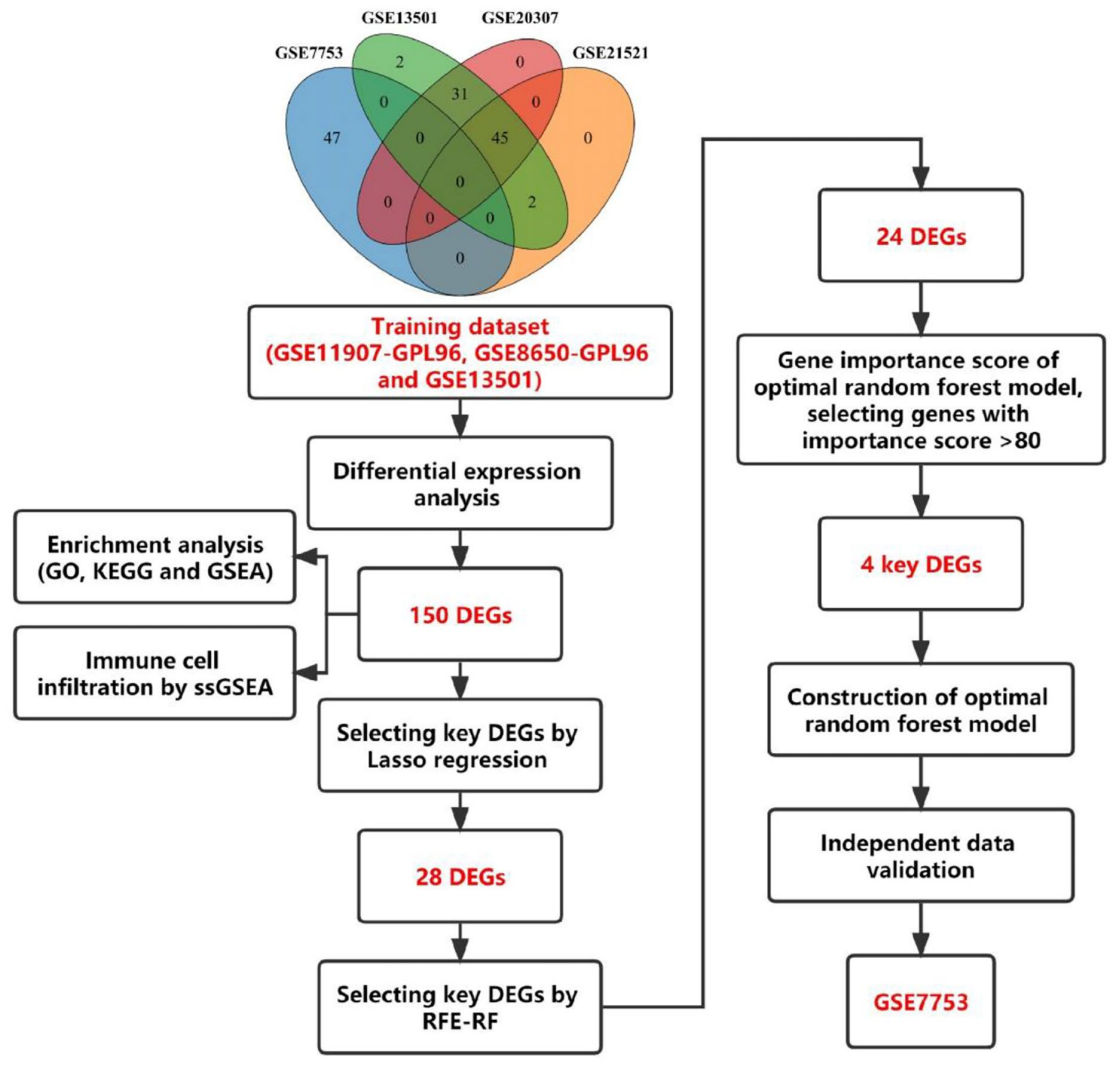
The flow chart of this study

Step 1: Since GSE7753, GSE13501, GSE20307 and GSE21521 are from the same institution (Cincinnati Childrens Hospital Medical Center), we extracted the sample numbers and performed intersection, and found that some of the samples among GSE13501, GSE20307 and GSE21521 are common to each other, where GSE13501 contains all the samples of GSE20307 and GSE21521. And GSE7753 is independent of these three datasets. Step 2: For the screening of DEGs, three datasets, GSE11907-GPL96, GSE8650-GPL96, and GSE13501, were pooled and used as training datasets as well as for differential expression analysis. Step 3: We performed GO, KEGG and GSEA analyses as well as analysis of immune cell infiltration. Step 4: We screened the key genes with a 10-fold cross-validation optimized Lasso. Step 5: We screened out the key genes with RF-RFE. Step 6: For the above key genes to be further optimized and screened, we used the optimal random forest model to score the gene importance and screen out 4 key genes. Step 7: Optimal random forest models were developed based on the key genes. Step 8: Further model validation was performed using the GSE7753 dataset. Figure 1 depicts the entire study flow.

### Identification of DEGs

**Fig. 2:**
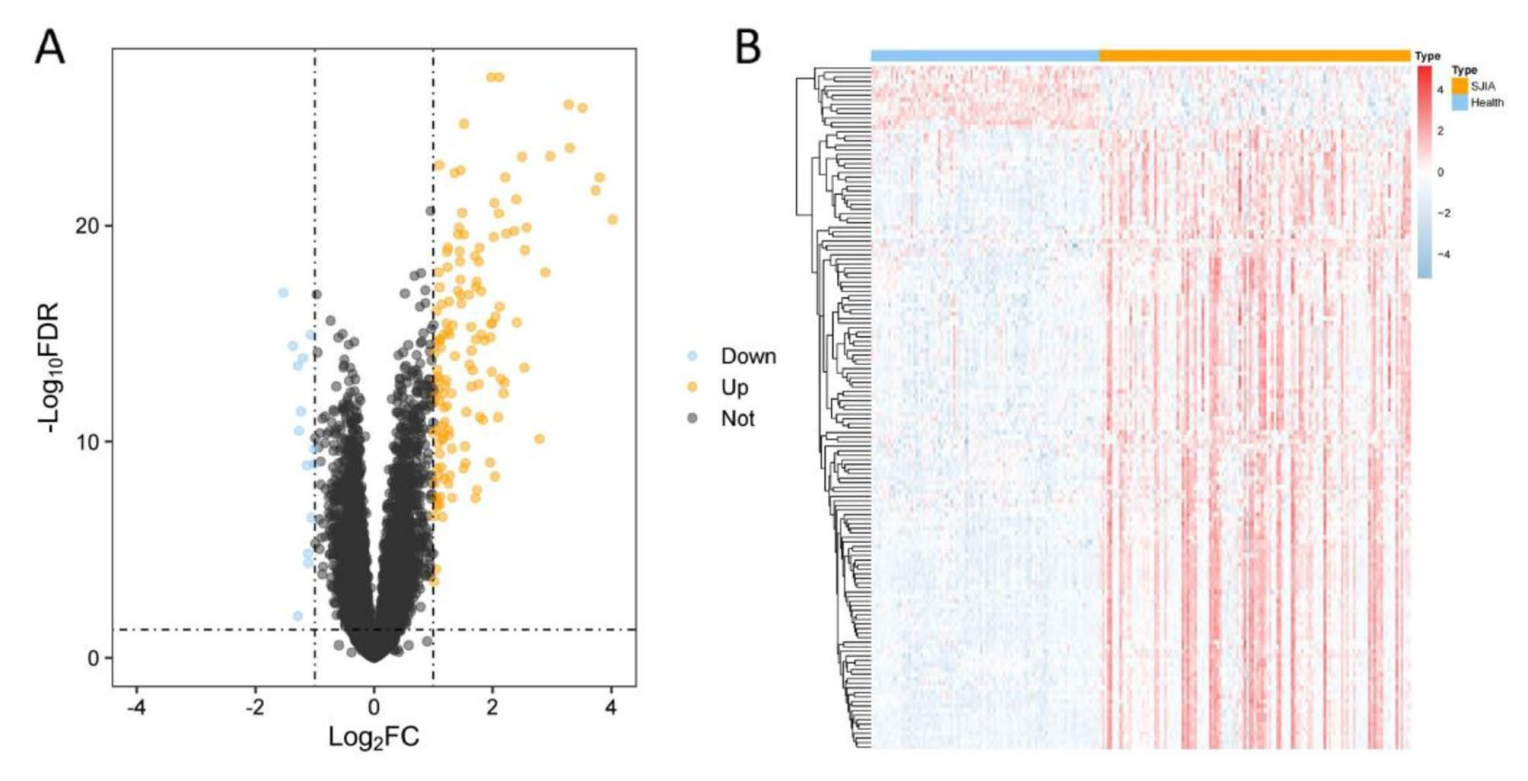
Differential genes. (**A**) Volcano map with 22 DEGs, orange dots indicate up-regulated genes, black dots indicate non-differentiated genes, and blue dots indicate down-regulated genes. (**B**) Heat map of the expression of 150 DEGs.

We performed differential expression analysis to screen DEGs in the merged training dataset, where there were 125 SJIA samples and 92 healthy samples in the training dataset. Using the significance criteria for DEGs, we identified 150 DEGs associated with SJIA, including 14 down-regulated genes and 136 up-regulated genes, and used a volcano plot to depict the expression status of all DEGs (Fig. 2A). By heat map, we likewise found that the expression levels of DEGs in SJIA, compared to healthy samples, were significantly different (Fig. 2B).

### Enrichment analysis

**Fig. 3:**
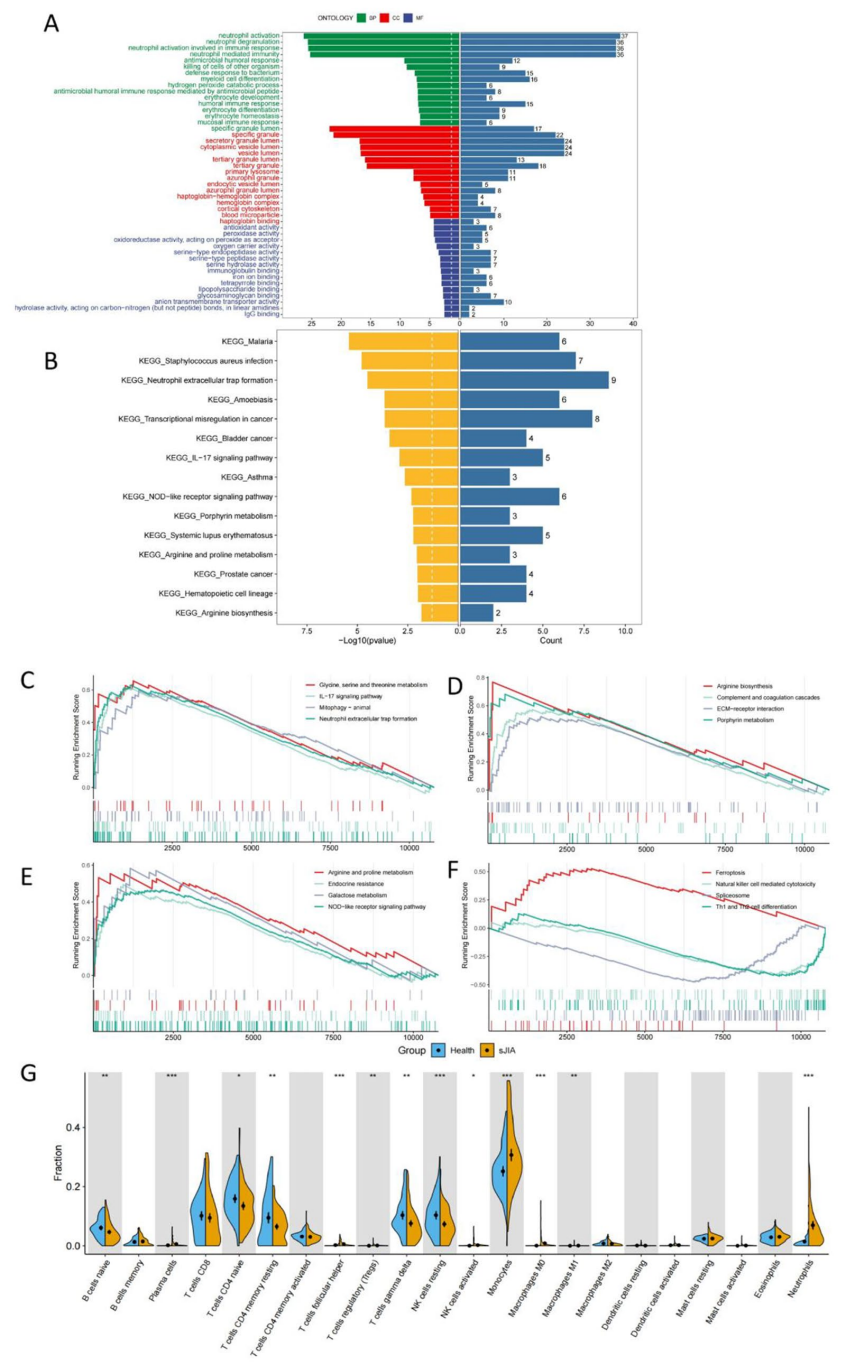
Enrichment analysis and immune cell infiltration analysis. (**A**) Histogram of GO enrichment analysis. (**B**) Histogram of KEGG enrichment analysis. (**C**-**D**) About pathway-related GSEA. (**G**) Violin plots of the 22 immune cell abundance differences analyzed between SJIA and healthy groups. (*p < 0.05; **p < 0.01; ***p < 0.001)

Then, enrichment analyses were performed for these DEGs. As shown by the results of GO analysis (Fig. 3A), DEGs were significantly enriched in immune and erythrocyte-related biological processes, such as neutrophil activation, humoral immune response and erythrocyte development. According to the results of KEGG analysis (Fig. 3B), immune-related disease pathways, IL-17 signaling pathway, NOD-like receptor signaling pathway, Porphyrin metabolism and Hematopoietic cell lineage were mainly enriched. By using GSEA, we further investigated the changes in pathways between SJIA and healthy samples (Fig. 3C-F), which revealed that SJIA patients were enriched for many metabolism- and immune-related pathways as well as two pathways associated to cell death (Mitophagy and Ferroptosis). Finally, we explored the immune differences between SJIA and healthy samples (Fig. 3G), and the results showed that compared with healthy samples, B cells naive, T cells CD4 naive, T cells CD4 memory resting, T cells gamma delta and NK cells in SJIA patients resting showed a significant down-regulation trend, while Plasma cells, T cells follicular helper, T cells regulatory (Tregs), Monocytes, Macrophages M0, Macrophages M1 and Neutrophils showed a significant up-regulation trend.

### Screening for key genes

**Fig. 4:**
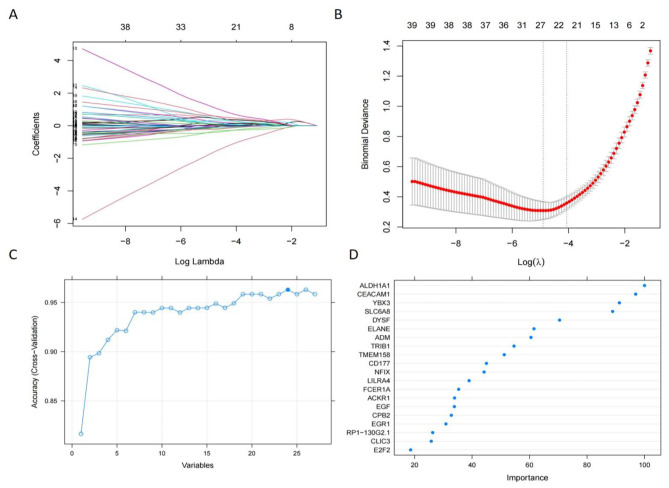
Feature selection. (**A**) The lasso regression curve of 28 DEGs. (**B**) The 10-fold cross-validation parameter (λ) options. (**C**) The 10-fold cross-validation of accuracy of signature gene combination of RF-RFE. (**D**) Gene importance scores for RF classifier

To obtain the key genes, we first entered all 150 DEGs into Lasso regression and performed 10 cross-validations. Based on the Lambda value at the minimum Binomial Deviance as a criterion (Fig. 4A-B), we identified 28 candidate genes by compressing the feature variables. Secondly, we utilized the RF-RFE method for re-feature selection, as shown in Fig. 4C, and it could be found that the model had the highest accuracy with 24 candidate genes. Finally, we incorporated 24 candidate genes into the random forest classifier and repeated 10-fold cross-validation 5 times to obtain the best model. To reduce the number of feature variables while preserving good predictive power, we identified 4 genes with importance scores > 80 as the final key genes for further analysis. As shown in Fig. 4D, the key genes include ALDH1A1, CEACAM1, YBX3 and SLC6A8.

### Construction and validation of the SJIA random forest model

**Fig. 5:**
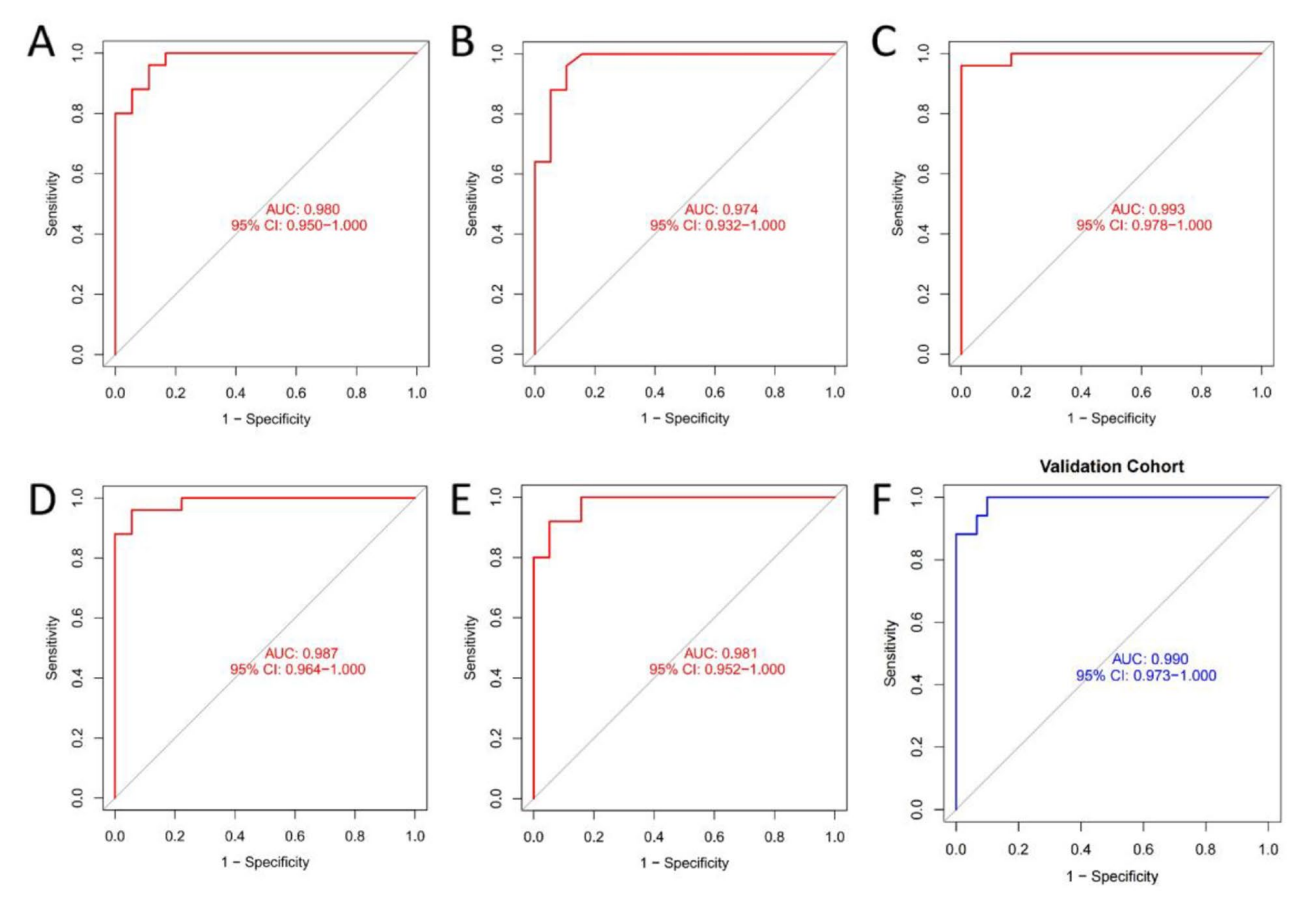
The ROC curve results for 5-fold cross-validation (**A**-**E**) and validation (**F**) dataset

We incorporated ALDH1A1, CEACAM1, YBX3 and SLC6A8 into the random forest classifier. To optimize the performance of the model, we performed a grid search for the mtry parameters and calculated the model accuracy for each mtry using 10-fold cross-validation with 5 repetitions. Finally, the highest accuracy of the random forest prediction model was locked at mtry = 2, and the optimal random forest prediction model was obtained. Subsequently, we performed a 5-fold cross-validation robustness test on the model, and each result was represented by a ROC curve (Fig. 5A-E), while the results for accuracy, Kappa, sensitivity, and specificity were shown in Table 1. The average AUC of the five cross-validation results was greater than 0.95, which proved the validity and robustness of the model. In the validation dataset, the ROC curve analysis estimated an AUC value of 0.990 (Fig. 5F), and the accuracy, Kappa, sensitivity and specificity of the confusion matrix estimation were 0.936, 0.864, 0.941 and 0.933, respectively, indicating the robustness of the model in predicting SJIA. Immediately after, we validated the predictive ability of the model for SLE, S. aureus, S. pneumoniae, E. coli and Influenza A. We found that their AUC values were 0.793, 0.834, 0.795, 0.913 and 0.755, respectively (Supplementary Table S4). It had a good predictive ability in E. coli, and the AUC of the other four diseases was less than 0.85, and the model was especially poor in predicting Influenza A. The model had a good predictive ability in E. coli. However, in the end, the model was the most effective in predicting SJIA.

**Table 1:** The results of 5-fold cross-validation

	AUC	Accuracy	Kappa	Sensitivity	Specificity
Cross validation 1	0.980	0.907	0.809	0.920	0.889
Cross validation 2	0.974	0.932	0.858	1.000	0.842
Cross validation 3	0.993	0.977	0.953	0.960	1.000
Cross validation 4	0.987	0.930	0.858	0.920	0.944
Cross validation 5	0.981	0.909	0.812	0.960	0.842

### Differential trends of the 4 key genes in the other three subtypes

**Fig. 6:**
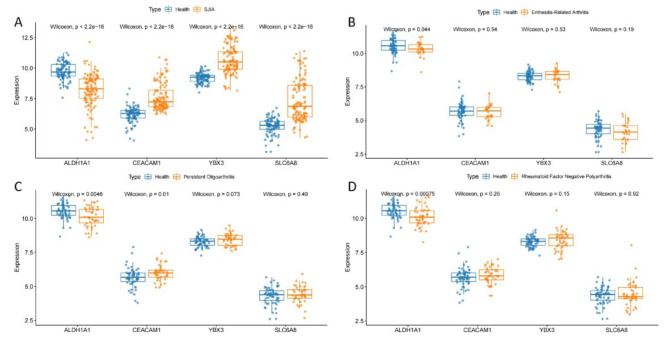
Differential expression of 4 key genes in SJIA, enthesitis-related arthritis, persistent oligoarthritis and rheumatoid factor negative polyarthritis

We further investigated 4 key genes, ALDH1A1, CEACAM1, YBX3 and SLC6A8, for differential trends among the 4 subtypes of JIA (Fig. 6A-D). It was found that ALDH1A1 showed a significant trend of down-regulation in all subtypes of JIA. CEACAM1 showed significant overexpression only in SJIA and persistent oligoarthritis. YBX3 and SLC6A8 showed significant overexpression in SJIA, while in the other three subtypes, neither gene showed differences compared to healthy individuals. Immediately after, in SJIA, we found by correlation analysis of 4 genes (Supplementary Fig. 1A) that there was a close association between the 4 genes, and we also found a strong positive correlation between YBX3 and SLC6A8 with a correlation coefficient of 0.92(Supplementary Fig. 1B).

### Analysis of GSEA, clinical phenotype and association with immune cells for 4 key genes

Our results from the GESA study of four key genes (Supplementary Table S5) showed that patients with low expression of ALDH1A1 were associated with cell cycle, neuroactive ligand-receptor interaction, ECM-receptor interaction, mucin type O-glycan biosynthesis, and glycine, serine and threonine metabolism. Patients with high CEACAM1 expression are associated with many immune-related pathways. Then, Patients with high SLC6A8 expression were associated with Glycine, serine and threonine metabolism, Porphyrin metabolism and Complement and coagulation cascades. The pathways associated with high YBX3 and SLC6A8 patients were very similar, further demonstrating a strong association between YBX3 and SLC6A8. In addition, patients with high CEACAM1 expression, high YBX3 expression, and high SLC6A8 expression all showed a strong connection with mitophagy, whereas patients with high YBX3 expression and high SLC6A8 expression showed a strong association with ferroptosis.

We used the clinical phenotypes provided by the GSE8650 to investigate the association between the four key genes and the clinical phenotypes. The differences between the four key genes across clinical phenotypes are illustrated by box plots (Supplementary Figure S2 A-D). In ALDH1A1, the lower the expression, the more likely it was that arthritis was accompanied by fever compared to asymptomatic patients. In CEACAM1, YBX3 and SLC6A8, all three genes were found to be overexpressed in patients with arthritis associated with fever.

In a further analysis of the correlation between the four key genes and immune cells, we used the spearman method for correlation analysis (Supplementary Figure S2 E) and showed that ALDH1A1 was positively correlated with T cells CD4 memory resting, Dendritic cells resting and negatively correlated with T cells CD4 memory activated, Neutrophils. In contrast, CEACAM1, YBX3 and SLC6A8 were found to be negatively correlated with T cell CD4 memory resting and positively correlated with T cell CD4 memory activated, Neutrophils.

## Discussion

Arthritis in SJIA is accompanied by clinical features such as fever, lymphadenopathy, rash, and serosal disease, and it develops rapidly and is prone to disability [1]. In order to enhance and protect the quality of survival for children with SJIA, good prediction and identification are essential [9]. However, the exact mechanism by which SJIA occurs is still unknown [26], and it is not a classic autoimmune disease, it has systemic inflammatory properties [6]. Since there are currently no clear diagnostic criteria for SJIA, the diagnosis is typically made using a combination of clinical symptoms, serological tests, and imaging studies. As a rare disease [27, 28], timely judgments are difficult to made in clinical diagnosis. On the genetic side, exploratory studies on biomarkers of SJIA are still of great value to go deeper. Therefore, it is essential to identify biomarkers that have a strong correlation with SJIA.

In our study, we used transcriptomic data from PBMC and built a SJIA diagnostic model based on the random forest algorithm as a way to distinguish between children with SJIA and healthy children. With the rapid development of bioinformatics, the evidence for the classification of diseases such as SJIA can be supported by strong evidence. To identify differential genes in SJIA, we first combined three GEO datasets (GSE11907-GPL96, GSE8650-GPL96 and GSE13501) as a training dataset, and then performed differential gene expression analysis. GO, KEGG and GSEA enrichment analyses were then performed. Based on GO and KEGG analyses, DEGs were associated with a large number of biological processes and pathways, reflecting the dynamic and complex nature of the pathogenesis. Many previous studies have supported our findings. Although children with SJIA and other JIA have long-term joint damage, the main difference lies in the fact that it can show autoinflammatory characteristics, which is closely related to autoimmunity. Brown et al. [29] found that neutrophils in children with SJIA exhibited sustained pro-inflammatory activation regardless of disease activity.IL-17, a pro-inflammatory cytokine secreted by Th17 cells, is also critical to study in SJIA. Feng et al. [30] study found that IL-17 levels were increased in both the active and inactive phases of SJIA compared to controls and were significantly higher in the active phase compared to the inactive phase. Omoyinmi et al. [31] reported a significant increase in the proportion of IL-17-producing CD4 + T cells in the peripheral blood of SJIA patients. Our study also found that differential genes associated with SJIA are linked to several metabolism-related pathways, including “Porphyrin metabolism”, “Arginine and proline metabolism” and “Glycine, serine and threonine metabolism”. GSEA analysis is superior to KEGG analysis and provides a better understanding of the biological changes between groups. The aggregation of many immune-related pathways in SJIA patients suggests that the pathogenesis of SJIA patients is associated with immune abnormalities, which has become a consensus among SJIA researchers. Regarding metabolism-related pathways, these pathways were positively correlated with SJIA, again suggesting that the pathogenesis of SJIA is likely to be closely related to metabolic dysregulation. We also found two cell death modalities, mitophagy and ferroptosis, to be significantly enriched in SJIA. Mitophagy is an important form of autophagy used to selectively remove dysfunctional or redundant mitochondria, and there is growing evidence of a close relationship between mitophagy and inflammation and autoimmunity [32–34]. Because most of the relevant studies are from autophagy-mitophagy-inhibition models, it should be noted that excessive activation of mitophagy may be detrimental to immune cell homeostasis, as Jin et al. [35] found that excessive activation of mitophagy performed in a mouse model impairs progenitor-phase red lineage differentiation and leads to depletion of red lineage cells. Therefore, the relationship between regulation of mitophagy and inflammation and immunity cannot be conclusively established, and multiple methods to assess activity of mitophagy are still needed to gain a more thorough understanding of pathways of mitophagy in autoimmune diseases [32]. Our study found that mitophagy was enriched in children with SJIA, which may provide new clues to future researchers about the relationship between SJIA and mitophagy. Ferroptosis is a regulated mode of cell death triggered by a combination of iron toxicity, lipid peroxidation, and plasma membrane damage [36]. Recent studies have identified an important role for this mode of cell death in metabolism and immunity [37, 38], while it has been less studied in SJIA.

We finally screened 4 key genes, namely ALDH1A1, CEACAM1, YBX3 and SLC6A8, by three feature screening methods (Lasso, RF-RFE and RF classifier scoring). Acetaldehyde dehydrogenase 1A1 (ALDH1A1) has a key role in tumor immunity. Liu et al. [39] found that increased ALDH1A1 activity in breast cancer led to expansion of myeloid-derived suppressor cells and immunosuppression, and also found that the ALDH1A1 inhibitor disulfiram and the chemotherapeutic agent gemcitabine synergistically inhibited breast tumor growth and tumorigenesis by clearing ALDH + tumor-initiating cells and activating T-cell immunity. Cui et al. [40] reported that ALDH1A1 expression in thyroid cancer was negatively correlated with immunostimulatory genes, major histocompatibility complexes, chemokines and receptors. The above two studies indicate that ALDH1A1 has a close relationship with immunity, but ALDH1A1 has been less studied in autoimmune diseases. In our study, we found that ALDH1A1 expression was low in children with SJIA compared to healthy children and that ALDH1A1 was positively correlated with T cell CD4 memory resting status and negatively correlated with T cell CD4 memory activation, consistent with previous reports that ALDH1A1 has an immunosuppressive function. A recent study reported that ALDH1A1 is associated with systemic sclerosis and affects the pentose phosphate pathway, oxidative stress and lipolysis [41], suggesting a relationship between ALDH1A1 and autoimmune diseases. Carcinoembryonic antigen cell adhesion molecule 1 (CEACAM1) plays an important role in regulating immune responses associated with infection, inflammation, and cancer. Previous studies have shown that CEACAM1 is a heterophile ligand of TIM-3, mediating T cell inhibition. The interaction between CEACAM1 and TIM-3 plays a crucial role in regulating autoimmunity and anti-tumor immunity [42, 43]. Our study found that CEACAM1 was highly expressed in children with SJIA and that patients with high CEACAM1 expression were associated with many immune-related pathways. CEACAM1 expression was positively correlated with plasma cells, T cells CD4 memory activation and neutrophils, suggesting that CEACAM1 may play a role in immune activation in SJIA. Y-box binding protein 3 (YBX3) is an RNA-binding protein that regulates different sets of mRNAs, including mRNA abundance, through different mechanisms, thus becoming a key regulator at the amino acid level [44]. It is considered a key molecule that links the genetic risk factors for Behcet syndrome to the pathogenesis of the disease [45]. Sun et al. [46] identified lncRNA-HEIH/YBX3 as a pan-cancer immunogenic system that can be used as a diagnostic and prognostic biomarker and therapeutic target. YBX3 is less studied at present, especially in autoimmune diseases. We found that YBX3 was significantly expressed in the vast majority of immune cells by single-cell level analysis of PMBC from 21 SJIA patients and 5 healthy individuals (Supplementary Figure S3). Alteration of macrophage-mediated immune response in vivo by depletion of intracellular creatine through ablation of creatine transporter protein (SLC6A8) [47]. SLC6A8 regulates the energy balance of intestinal epithelial cells, thereby regulating intestinal epithelial integrity and barrier function, which leads to intestinal barrier dysfunction in patients with inflammatory bowel disease [48]. We found that YBX3 and SLC6A8 were highly expressed in SJIA and that there was a strong correlation between YBX3 and SLC6A8. The results of GSEA analysis, clinical phenotype analysis and correlation analysis with immune cells for these two genes showed strong similarity. In the GSEA study, high expression was found to be closely associated with Complement and coagulation cascades. In a correlation analysis, they were found to be negatively correlated with T cells CD4 memory resting state and positively correlated with T cells CD4 memory activation state, and these two genes may play a key role in the regulation of the immune system in SJIA. Currently, these 4 genes have not been studied in SJIA. In this study, we performed a key gene screen using machine learning techniques with public genetic databases, and we suggest that these 4 genes may be key biomarkers linking genetic risk factors for SJIA to disease pathogenesis.

In the study, we analysed three other subtypes of JIA (enthesitis-related arthritis, persistent oligoarthritis and rheumatoid factor negative polyarthritis) in relation to the expression of 4 key genes. ALDH1A1 showed a significant trend of low expression in all four subtypes of patients compared to healthy individuals, suggesting that ALDH1A1 plays a key role in JIA. In contrast, CEACAM1 showed high expression only in SJIA and persistent oligoarthritis patients, suggesting a potentially identical pathway between SJIA and persistent oligoarthritis. In contrast, YBX3 and SLC6A8 were only highly expressed in SJIA, with no difference in any of the other three subtypes, indicating the specificity of these two genes in SJIA compared to the other three subtypes.The highlights of our study are the innovative combination and application of Lasso, RF-RFE and Random Forest classifier scoring methods to SJIA and the excellent results generated in terms of predictive power in SJIA. Three feature selection methods, such as Lasso, RF-RFE and random forest classifier scoring, are each capable of being used as methods to identify key biomarkers and have been widely used in biology [49, 50]. Previously, no studies have used machine learning to construct SJIA diagnostic models based on gene sequencing.

We performed a 5-fold cross-validation to prevent the model from overfitting, and obtained an AUC greater than 0.95 and an accuracy greater than 0.9 in each case, indicating the robustness of the model. Following immediately, we performed another validation of the robustness and generalization ability of the model using an external independent dataset (GSE7753), and the results showed an AUC of 0.990, again demonstrating the strong robustness of our model. In particular, the sample in GSE7753 included untreated children with new-onset SJIA as well as healthy children. The above results indicate that the model is well suited for the identification and prediction of SJIA.

In addition, we explored whether there were similarities and differences among the 4 subtypes (SJIA, enthesitis-related arthritis, persistent oligoarthritis and rheumatoid factor negative polyarthritis) of these 4 key genes. The results revealed that ALDH1A1 showed downregulation in all 4 subtypes, and ALDH1A1 may have a key role as a biomarker of disease onset in the same 4 subtypes. YBX3 and SLC6A8 were only upregulated in SJIA, with no significant up and down expression trends in the remaining three subtypes, which may indicate that these two genes are the key genes that cause SJIA to have different clinical characteristics from the three subtypes. And at the same time YBX3 and SLC6A8 have a very strong correlation in SJIA.

Using the transcriptome level, we investigated the validity and reliability of machine learning in SJIA diagnostic prediction. In light of this, we have successfully developed a new SJIA disease risk prediction model that holds promise for use as a new SJIA diagnostic prediction tool to aid in the diagnosis of SJIA. Furthermore, the 4 key genes we finally obtained provided some new clues for the subsequent SJIA study.

Nevertheless, our study has some limitations. (1) Although we merged multiple datasets into a larger dataset to build the model, it still did not satisfy the number of data samples required for machine learning. If conditions allow, we can include more research data in future training datasets. (2) Overfitting in model construction is objective and difficult to eliminate, but we minimized the overfitting problem by using a 5-fold cross-validation approach in the modeling process. Checking for overfitting is not a complete solution, but it is still very helpful. However, this means that even if we get good model results on the validation dataset, there is no shortage of different data with noise in reality, and the actual generalization ability may not be good. (3) The model has not been tested in practical applications in predicting SJIA patients. Therefore, more research data will be needed in the future to test the robustness and generalization ability of the model.

## Conclusions

In conclusion, we explored and identified the key biomarkers ALDH1A1, CEACAM1, YBX3 and SLC6A8, which are significantly associated with SJIA, for the first time by using machine learning techniques through an in-depth study of the SJIA datasets in the GEO database, and the combination of these 4 genes can effectively construct a random forest-based SJIA diagnostic model as well as a powerful prediction of SJIA.

### Electronic supplementary material

Below is the link to the electronic supplementary material.

Supplementary Material 1

Supplementary Material 2

Supplementary Material 3

## Data Availability

The datasets supporting the conclusions of this article are available in the GEO (https://www.ncbi.nlm.nih.gov/geo/). GEO accession numbers: GSE13501, GSE11907, GSE20307, GSE21521, GSE7753, GSE8650, GSE6269 and GSE207633.
