# Supplementary material for: Establishment and analysis of a novel diagnostic model for systemic juvenile idiopathic arthritis based on machine learning
Source: Pediatr Rheumatol Online J. 2024 Jan 19;22:18. doi: 10.1186/s12969-023-00949-x (PMC10797915; doi:10.1186/s12969-023-00949-x)
Supplement: Supplementary file 1 — Supplementary Material 1 [file 12969_2023_949_MOESM1_ESM.docx]

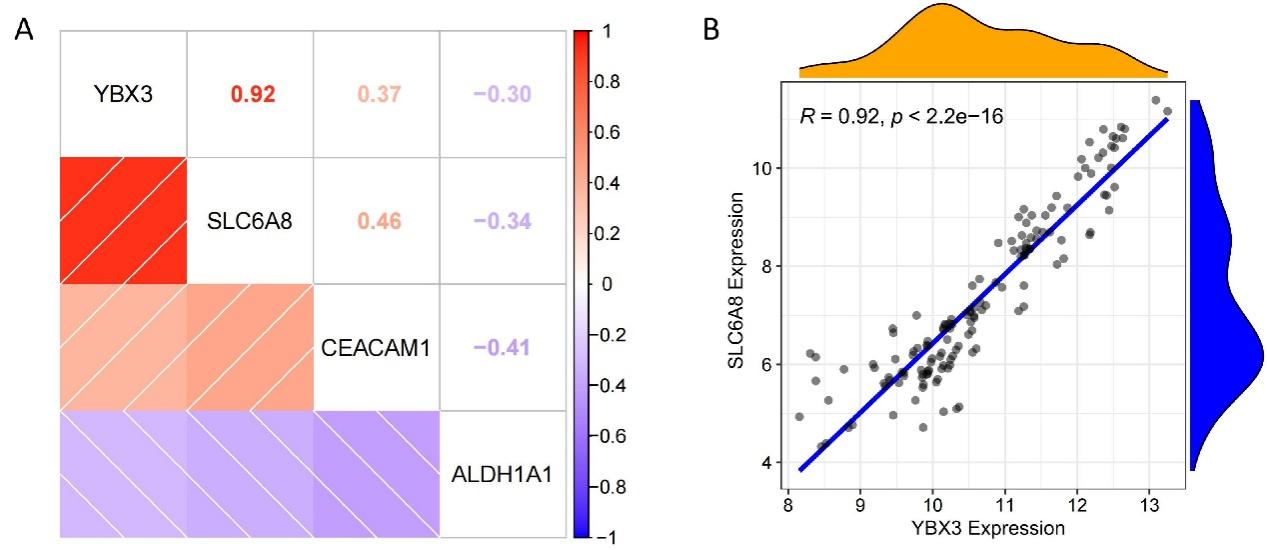


**Supplementary Figure S1** Correlation analysis. (A) Correlation between ALDH1A1, CEACAM1, YBX3 and SLC6A8 in SJIA. (B) Scatter plot of correlation between YBX3 and SLC6A8


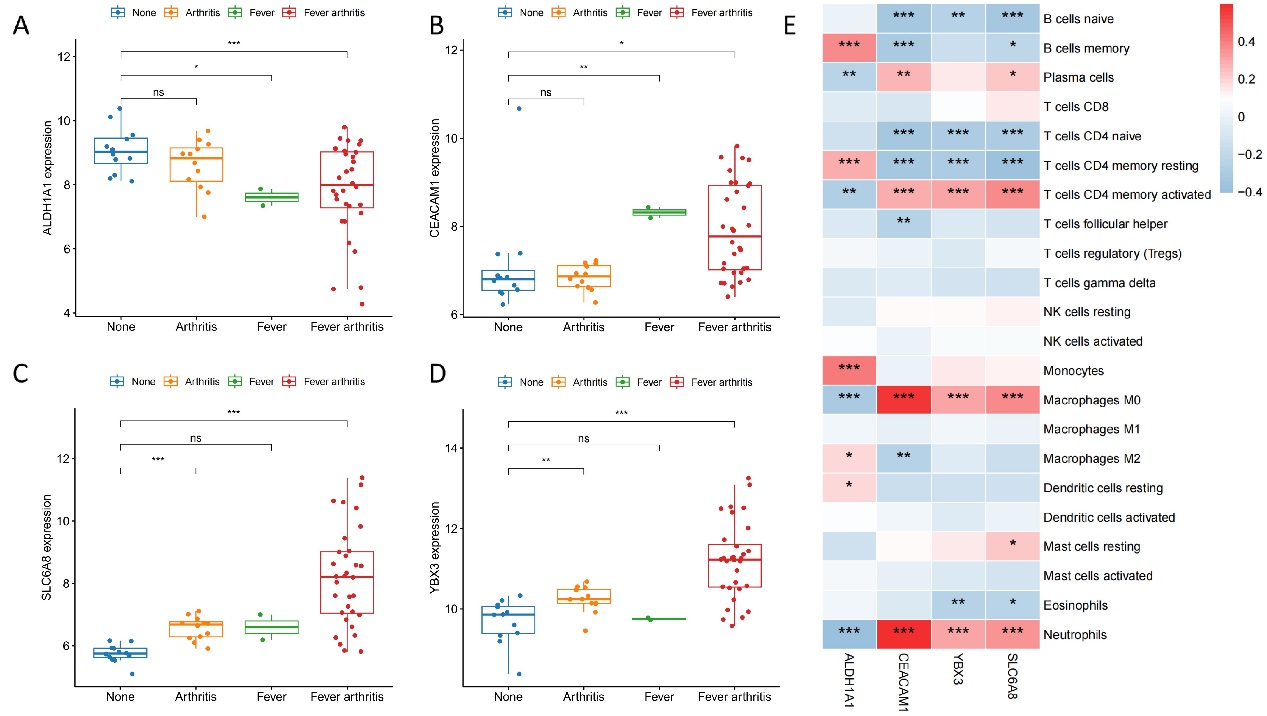


**Supplementary Figure S2** Clinical phenotypic analysis of 4 genes and their correlation with immune cells. (A) Analysis of the differences between ALDH1A1, CEACAM1, YBX3 and SLC6A8 and clinical symptoms in SJIA patients. (B) Heat map of the correlation analysis between ALDH1A1, CEACAM1, YBX3 and SLC6A8 and immune cells. (*p < 0.05; **p < 0.01; ***p < 0.001; ns, not significant).


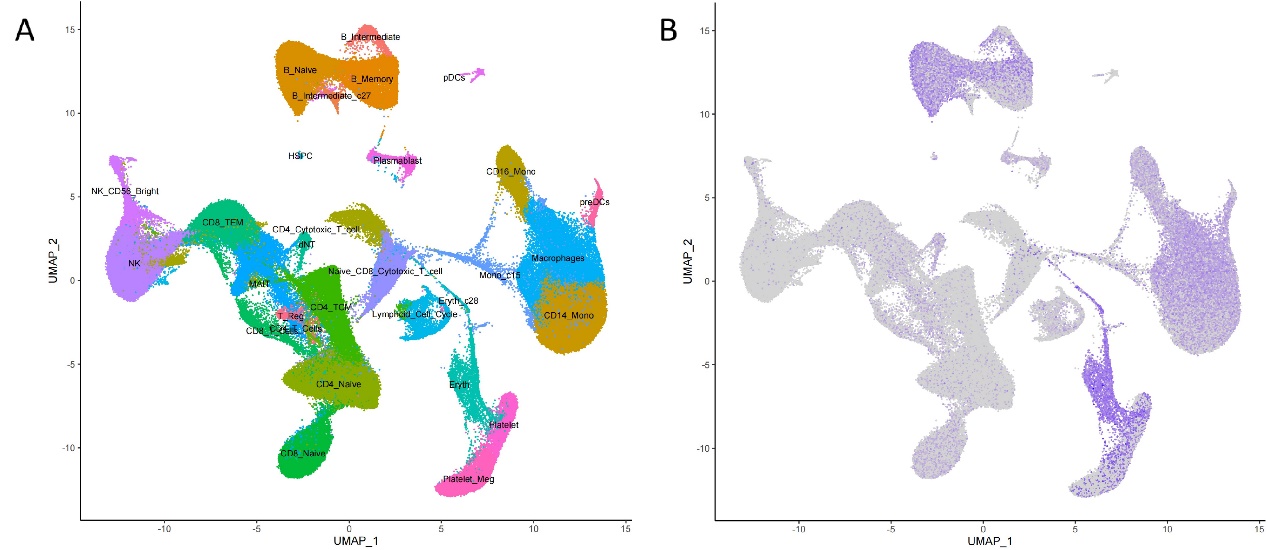


**Supplementary Figure S3** Distribution of immune cell types (A) at the single-cell level in the GSE207633 dataset as well as feature plot (B) of YBX3 expression levels in different immune cell types colored with YBX3 expression levels.
