# Supplementary material for: Establishment and analysis of a novel diagnostic model for systemic juvenile idiopathic arthritis based on machine learning
Source: Pediatr Rheumatol Online J. 2024 Jan 19;22:18. doi: 10.1186/s12969-023-00949-x (PMC10797915; doi:10.1186/s12969-023-00949-x)
Supplement: Supplementary file 2 — Supplementary Material 2 [file 12969_2023_949_MOESM2_ESM.docx]

**Supplementary Table S1 The information on the SJIA datasets of the GEO.**

| **GEO accession** | **Tissue** | **SJIA** | **Normal** | **Total** | **Group** |
| --- | --- | --- | --- | --- | --- |
| GSE11907-GPL96 | PBMCs | 46 | 12 | 58 | Training |
| GSE8650-GPL96 | PBMCs | 58 | 21 | 79 | Training |
| GSE13501 | PBMCs | 21 | 59 | 80 | Training |
| GSE20307 | PBMCs | 20 | 56 | 76 | - |
| GSE21521 | PBMCs | 18 | 29 | 47 | - |
| GSE7753 | PBMCs | 17 | 30 | 48 | Validation |

**Supplementary Table S2 The information on the GSE13501 of the GEO.**

| **GEO accession** | **Disease type** | **Tissue** | **Disease** | **Normal** | **Total** |
| --- | --- | --- | --- | --- | --- |
| GSE13501 | persistent oligoarthritis | PBMCs | 42 | 59 | 101 |
| GSE13501 | rheumatoid factor negative polyarthritis | PBMCs | 45 | 59 | 104 |
| GSE13501 | enthesitis-related arthritis | PBMCs | 28 | 59 | 87 |

**Supplementary Table S3 The information on the GSE8650 and GSE6269 of the GEO.**

| **GEO accession** | **Disease type** | **Tissue** | **Disease** | **Normal** | **Total** |
| --- | --- | --- | --- | --- | --- |
| GSE8650-GPL96 | SLE | PBMC | 38 | 21 | 59 |
| GSE6269-GPL96 | S. aureus | PBMC | 19 | 21  (GSE8650- GPL96) | 40 |
| GSE6269-GPL96 | S. pneumoniae | PBMC | 13 | 21  (GSE8650- GPL96) | 34 |
| GSE6269-GPL96 | E. coli | PBMC | 29 | 21  (GSE8650- GPL96) | 50 |
| GSE6269-GPL96 | Influenza A | PBMC | 18 | 21  (GSE8650- GPL96) | 39 |

**Supplementary Table S4 On the predictive power of models for** **SLE, S. aureus, S. pneumoniae, E. coli and Influenza A**

| Disease | AUC | Accuracy | Kappa | Sensitivity | Specificity |
| --- | --- | --- | --- | --- | --- |
| SLE | 0.793 | 0.763 | 0.482 | 0.816 | 0.667 |
| S. aureus | 0.834 | 0.827 | 0.654 | 0.774 | 0.905 |
| S. pneumoniae | 0.795 | 0.824 | 0.626 | 0.769 | 0.857 |
| E. coli | 0.913 | 0.880 | 0.754 | 0.897 | 0.857 |
| Influenza A | 0.755 | 0.744 | 0.492 | 0.833 | 0.667 |
